# Synthesis and crystal structure of 1-hy­droxy-8-methyl-9*H*-carbazole-2-carbaldehyde

**DOI:** 10.1107/S2056989021007210

**Published:** 2021-07-16

**Authors:** Aravazhi Amalan Thiruvalluvar, M. Sridharan, K. J. Rajendra Prasad, M. Zeller

**Affiliations:** a Principal (Retired), Kunthavai Naacchiyaar Government Arts College for Women (Autonomous), Thanjavur 613 007, Tamilnadu, India; b Department of Chemistry, RV College of Engineering, Bangalore 560 059, Karnataka, India; c Department of Chemistry, Bharathiar University, Coimbatore 641 046, Tamilnadu, India; d Department of Chemistry, Purdue University, West Lafayette, IN 47907-2084, USA

**Keywords:** crystal structure, 9*H*-carbazole, π–π stacks, N—H⋯O, O—H⋯O, C—H⋯O hydrogen bonding

## Abstract

Two crystallographically independent mol­ecules are present in the asymmetric unit of the title compound, C_14_H_11_NO_2_, with virtually identical geometries. The carbazole units are planar. The hy­droxy group at position 1, carbaldehyde group at position 2, and methyl group at position 8 (with the exception of two H atoms) are coplanar with the attached benzene rings. The dihedral angle between the two benzene rings is 2.20 (9)° in mol­ecule *A* and 2.01 (9)° in mol­ecule *B*. The pyrrole ring makes dihedral angles of 0.82 (10) and 1.40 (10)° [0.84 (10) and 1.18 (10)° in mol­ecule *B*] with the (–CH_3_)-substituted and (–OH and –CHO) substituted benzene rings, respectively. The mol­ecular structure is stabilized by the intra­molecular O—H⋯O hydrogen bonds, while the crystal structure features N—H⋯O and C—H⋯O hydrogen bonds. A range of π–π contacts further stabilizes the crystal structure.

## Abstract 2

Two crystallographically independent mol­ecules are present in the asymmetric unit. O—H⋯O, N—H⋯O and C—H⋯O hydrogen bonds form rings and chains and π–π stacks further connect mol­ecules in the crystal.

## Chemical context   

Nitro­gen-containing heterocyclic compounds are key building blocks used to develop chemicals of biological and medicinal inter­est. Among nitro­gen heterocycles, carbazole alkaloids represent an important class of natural products. The Indian medicinal plant *Murraya koenigii spreng* (Rutaceae) is a rich source of carbazole alkaloids (Knölker & Reddy, 2002 ▸), and a number of these natural products with novel structures and useful biological activities have been isolated from this plant over the past decades. The increase of isolable natural products as well as the pharmacological action of these carbazole derivatives has generated synthetic inter­est; consequently, the synthesis of carbazoles has been an active area of study.

**Figure 1:**
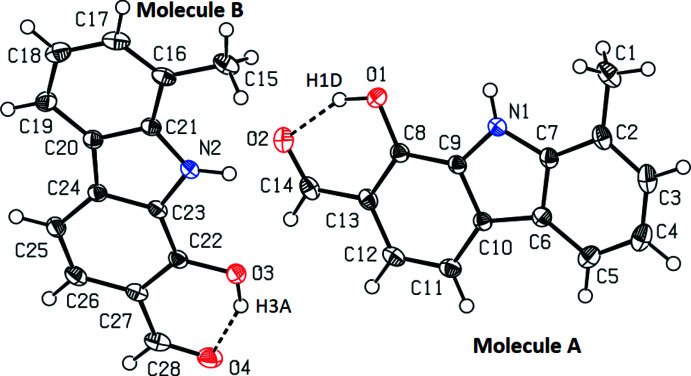
The two crystallographically independent mol­ecules with the atom-numbering scheme. Non-H atoms are shown at the 50% displacement ellipsoid probability level, H atoms are represented as small spheres.

Based on the structural, biological and pharmacological importance of carbazole derivatives, the present investigation was to devise a viable synthetic route to these compounds using different methodologies. For our synthetic strategy, 2,3,4,9-tetra­hydro-1*H*-carbazol-1-ones prepared in our laboratory were used as precursors, opening new avenues for the synthesis of highly functionalized carbazole derivatives such as 1-hy­droxy­imino-2,3,4,9-tetra­hydro-1*H*-carbazoles, 1-hy­droxy­carbazoles, and 1-hy­droxy-2-formyl­carbazoles. The functionalized carbazoles thus prepared lead to mukonine isomers, oxazolocarbazoles, girinimbine isomers, pyran­ocarbazoles, indoloisoflavones, indolocoumarins, indoloxanthones, benzocarbazoles, car­baz­ol­yl­oxy­prop­an­ol­amines and pyrazolo-, isoxazolo-, furo-, oxazino-, pyrimido-, pyridazino-, pyrido-, pyrazino- and indolo-carbazoles in excellent yields (Shanmugasundaram & Rajendra Prasad, 1999 ▸; Sridharan & Rajendra Prasad, 2011 ▸; Sridharan, Beagle *et al.*, 2008 ▸ and references cited therein). Herein, we report the synthesis and crystal structure of 1-hy­droxy-8-methyl-9*H*-carbazole-2-carbaldehyde (Fig. 1 ▸), which is a potential precursor for the synthesis of many hetero-annulated carbazoles (Gunaseelan *et al.*, 2007 ▸).
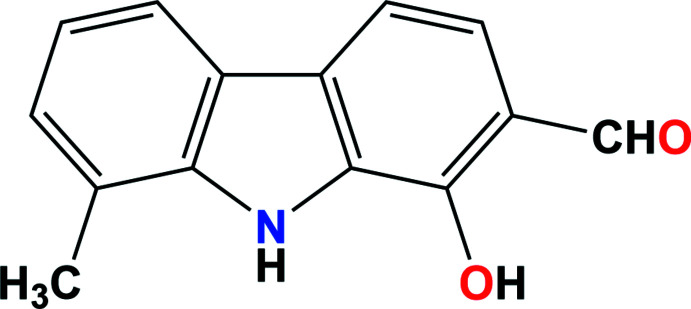


## Structural commentary   

**Figure 2:**
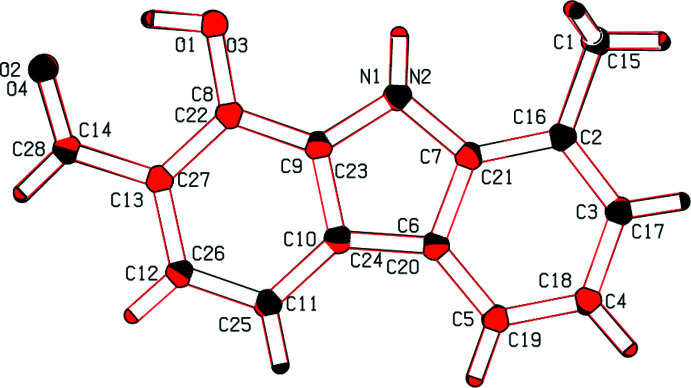
Least-squares overlay of the two independent mol­ecules (inverted mol­ecule *B* on mol­ecule *A*). Fit rotation angle is −172.76°, r.m.s. fit = 0.087 Å.

The title compound crystallizes in the monoclinic space group *P*2_1_/*c* with two independent mol­ecules (*A* and *B*, Fig. 1 ▸) in the asymmetric unit. They are superimposable and both are essentially planar. Placing inverted mol­ecule *B* on mol­ecule *A* gives the best fit, with the overlay of the two independent mol­ecules shown in Fig. 2 ▸. The weighted r.m.s. fit of the 17 non-H fitted atoms is 0.034 Å, the r.m.s. bond fit is 0.003 Å and the r.m.s. angle fit is 0.383°. Both independent mol­ecules, including the hy­droxy group at position 1, carbaldehyde group at position 2, and methyl group at position 8 (with the exception of two H atoms) are near planar. The dihedral angle between the two benzene rings of the carbazole is 2.20 (9)° in mol­ecule *A* and 2.01 (9)° in mol­ecule *B*. The pyrrole ring makes dihedral angles of 0.82 (10) and 1.40 (10)° for mol­ecule *A* and 0.84 (10) and 1.18 (10)° for mol­ecule *B* with the methyl-substituted and hydroxide/carbaldehyde-substituted benzene rings, respectively. The compound exhibits intra­molecular O—H⋯O hydrogen bonding between the hydroxide and aldehyde groups (Table 1 ▸). Hydrogen bonds similar to the O1—H1*D*⋯O2 and O3—H3*A*⋯O4 bonds observed in this structure, forming *S*(6) ring motifs, have previously been observed (Bernstein *et al.*, 1995 ▸).

## Supra­molecular features   

**Figure 3:**
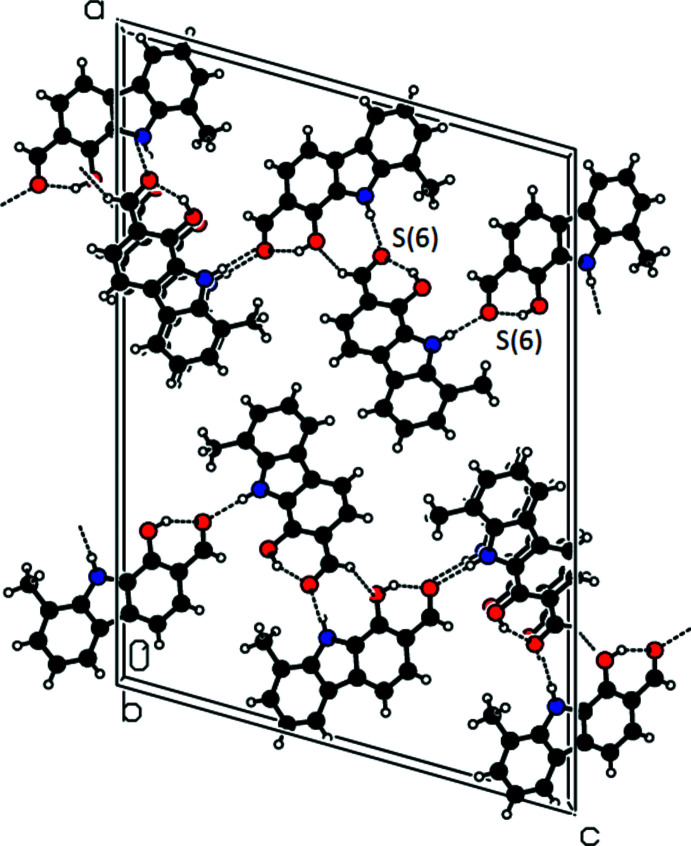
Perspective partial packing view of the title compound, viewed along the *b* axis, showing the hydrogen-bonding inter­actions. Black dashed lines indicate the N—H⋯O, O—H⋯O and C—H⋯O hydrogen bonds.

**Figure 4:**
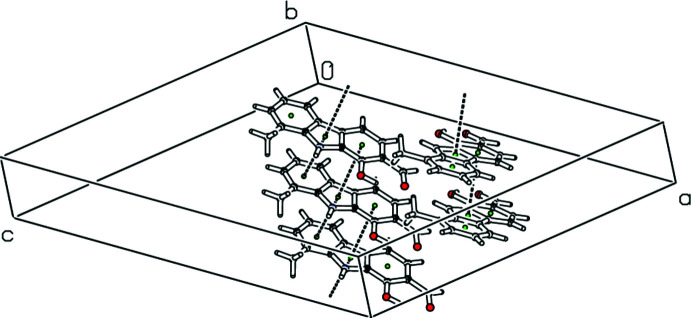
Straw-style packing view of the title compound, viewed down the *b* axis, showing slipped π–π stacking inter­actions. Centroids are indicated by green spheres and contacts between centroids by black dotted lines.

In the crystal, mol­ecules are connected into chains parallel to the *c* axis by inter­molecular N—H⋯O and C—H⋯O hydrogen bonds (Table 1 ▸, Fig. 3 ▸). Both crystallographically independent mol­ecules are arranged in similar N1—H1⋯O4(*x*, 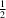 − *y*, 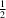 + *z*) and N2—H2⋯O2(*x*, 1 + *y*, *z*) hydrogen bonds. A C14—H14⋯O3(*x*, −1 + *y*, *z*) hydrogen bond is also present. A range of π–π contacts is also observed (Fig. 4 ▸). The distances between ring centroids are *Cg*1⋯*Cg*2(*x*, −1 + *y*, *z*) = 3.4604 (13) Å, *Cg*1⋯*Cg*3 (*x*, 1 + *y*, *z*) = 3.4896 (13) Å and *Cg*7⋯*Cg*9 (*x*, 1 + *y*, *z*) = 3.6279 (13) Å, where *Cg*1, *Cg*2, *Cg*3, *Cg*7 and *Cg*9 are the centroids of the N1/C7/C6/C10/C9, C2–C7, C8–C13, N2/C21/C20/C24/C23 and C22–C27 rings, respectively.

## Database survey   

A search in the Cambridge Structural Database (CSD, Version 5.42, update May 2021; Groom *et al.*, 2016 ▸) for the structure 1-hy­droxy-8-methyl-9*H*-carbazole-2-carbaldehyde gave two hits, *viz*. 2,2,10-trimethyl-2,3-di­hydro­pyrano(2,3-a)carbazol-4(11*H*)-one (CSD refcode: BOGTOH; Sridharan, Prasad *et al.*, 2008 ▸) and 1-(1-hy­droxy-8-methyl-9*H*-carbazol-2-yl)ethanone (CSD refcode: WACYEG; Archana *et al.*, 2010 ▸). A search for the structure of 9*H*-carbazole-1-ol gave 69 hits. 1-Hy­droxy-3-methyl-9*H*-carbazole-2-carbaldehyde, C_14_H_11_NO_2_, (CSD refcode: NIFCUB; Gunaseelan *et al.*, 2007 ▸) has the most similar structure to that of the title compound, with a 3-methyl rather than an 8-methyl group. The structure of NIFCUB is similarly stabilized by inter- and intra­molecular N—H⋯O and O—H⋯O hydrogen bonds.

## Synthesis and crystallization   

**Figure 5:**
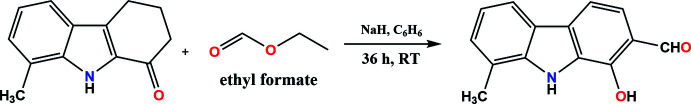
Synthesis of the title compound.

30% Sodium hydride in mineral oil (2.4 g) was washed with dry benzene and taken into a round-bottom flask containing dry benzene (100 ml). The flask was kept in an ice bath under stirring. Ethyl formate (8 ml) was added dropwise to the solution over a period of 10 minutes. Then 8-methyl-2,3,4,9-tetra­hydro-1*H*-carbazol-1-one (1.6 g, 0.008 mol) in dry benzene (25 ml) was added slowly and the reaction mixture was allowed to stir for another 36 h. The reaction was monitored by TLC. After completion of the reaction, benzene was removed *in vacuo* and the contents in the flask were transferred to a beaker containing water. It was neutralized with dilute HCl, filtered, washed with water and dried to get crude 1-hy­droxy-8-methyl-9*H*-carbazole-2-carbaldehyde. It was purified by column chromatography over silica using petroleum ether:ethyl acetate (95:5) as eluant. The brown pure product obtained was recrystallized using glacial acetic acid (needle-shaped crystals, yield 0.965 g, 55%), m.p. 414 K (Fig. 5 ▸).

## Refinement   

Crystal data, data collection and structure refinement details are summarized in Table 2 ▸. The indole NH hydrogen atoms, H1 and H2 and the hydroxyl OH hydrogen atoms H1*D* and H3*A* were located in a difference-Fourier map and freely refined. The remaining hydrogen atoms were placed in calculated positions with C—H bond distances of 0.93 Å (aromatic H), or 0.96 Å (methyl H) and were refined with anisotropic displacement parameters 1.2 and 1.5 times that of the parent carbon atoms.

## Supplementary Material

Crystal structure: contains datablock(s) I. DOI: 10.1107/S2056989021007210/yy2001sup1.cif

Structure factors: contains datablock(s) I. DOI: 10.1107/S2056989021007210/yy2001Isup2.hkl

Supporting information file. DOI: 10.1107/S2056989021007210/yy2001Isup3.cdx

Supporting information file. DOI: 10.1107/S2056989021007210/yy2001Isup4.cml

CCDC reference: 1540679

Additional supporting information:  crystallographic information; 3D view; checkCIF report

## Figures and Tables

**Table 1:** Hydrogen-bond geometry (Å, °)

*D*—H⋯*A*	*D*—H	H⋯*A*	*D*⋯*A*	*D*—H⋯*A*
C14—H14⋯O3^i^	0.93	2.50	3.254 (2)	138
N1—H1⋯O4^ii^	0.87 (2)	2.00 (2)	2.862 (2)	174 (2)
O1—H1*D*⋯O2	0.94 (3)	1.74 (3)	2.602 (2)	151 (3)
N2—H2⋯O2^iii^	0.91 (2)	1.97 (2)	2.879 (2)	173 (2)
O3—H3*A*⋯O4	0.90 (3)	1.78 (3)	2.595 (2)	150 (3)

Symmetry codes: (i) x, y-1, z; (ii) x, -y+{\script{1\over 2}}, z+{\script{1\over 2}}; (iii) x, y+1, z.

**Table 2:** Experimental details

Crystal data	
Chemical formula	C_14_H_11_NO_2_
*M*_r_	225.24
Crystal system, space group	Monoclinic, *P*2_1_/*c*
Temperature (K)	296
*a*, *b*, *c* (Å)	28.290 (5), 3.9052 (7), 20.264 (3)
β (°)	105.817 (2)
*V* (Å^3^)	2154.0 (6)
*Z*	8
Radiation type	Mo *K*α
μ (mm^−1^)	0.09
Crystal size (mm)	0.75 × 0.19 × 0.10

Data collection	
Diffractometer	Bruker SMART APEXII CCD
Absorption correction	Multi-scan (*SADABS*; Bruker, 2005 ▸)
*T*_min_, *T*_max_	0.830, 0.991
No. of measured, independent and observed [*I* > 2σ(*I*)] reflections	19760, 5344, 4453
*R*_int_	0.034
(sin θ/λ)_max_ (Å^−1^)	0.667

Refinement	
*R*[*F*^2^ > 2σ(*F* ^2^)], *wR*(*F* ^2^), *S*	0.055, 0.146, 1.18
No. of reflections	5344
No. of parameters	325
H-atom treatment	H atoms treated by a mixture of independent and constrained refinement
Δρ_max_, Δρ_min_ (e Å^−3^)	0.31, −0.24

Computer programs: *APEX2* and *SAINT* (Bruker, 2005 ▸), *SHELXTL* (Sheldrick, 2008 ▸), *SHELXL2018/3* (Sheldrick, 2015 ▸), *PLATON* (Spek, 2020 ▸) and *publCIF* (Westrip, 2010 ▸).

## supplementary crystallographic information

### Crystal data

**Table:**

C_14_H_11_NO_2_	*F*(000) = 944
*M**_r_* = 225.24	*D*_x_ = 1.389 Mg m^−^^3^
Monoclinic, *P*2_1_/*c*	Mo *K*α radiation, λ = 0.71073 Å
*a* = 28.290 (5) Å	Cell parameters from 7327 reflections
*b* = 3.9052 (7) Å	θ = 2.2–31.3°
*c* = 20.264 (3) Å	µ = 0.09 mm^−^^1^
β = 105.817 (2)°	*T* = 296 K
*V* = 2154.0 (6) Å^3^	Needle, brown
*Z* = 8	0.75 × 0.19 × 0.10 mm

### Data collection

**Table:**

Bruker SMART APEXII CCD diffractometer	5344 independent reflections
Radiation source: fine-focus sealed tube	4453 reflections with *I* > 2σ(*I*)
Graphite monochromator	*R*_int_ = 0.034
ω scans	θ_max_ = 28.3°, θ_min_ = 1.5°
Absorption correction: multi-scan (SADABS; Bruker, 2005)	*h* = −37→37
*T*_min_ = 0.830, *T*_max_ = 0.991	*k* = −5→5
19760 measured reflections	*l* = −26→26

### Refinement

**Table:**

Refinement on *F*^2^	Primary atom site location: structure-invariant direct methods
Least-squares matrix: full	Secondary atom site location: difference Fourier map
*R*[*F*^2^ > 2σ(*F*^2^)] = 0.055	Hydrogen site location: mixed
*wR*(*F*^2^) = 0.146	H atoms treated by a mixture of independent and constrained refinement
*S* = 1.18	*w* = 1/[σ^2^(*F*_o_^2^) + (0.0612*P*)^2^ + 1.1713*P*] where *P* = (*F*_o_^2^ + 2*F*_c_^2^)/3
5344 reflections	(Δ/σ)_max_ = 0.001
325 parameters	Δρ_max_ = 0.31 e Å^−^^3^
0 restraints	Δρ_min_ = −0.24 e Å^−^^3^

### Special details

**Table:**

Geometry. All esds (except the esd in the dihedral angle between two l.s. planes) are estimated using the full covariance matrix. The cell esds are taken into account individually in the estimation of esds in distances, angles and torsion angles; correlations between esds in cell parameters are only used when they are defined by crystal symmetry. An approximate (isotropic) treatment of cell esds is used for estimating esds involving l.s. planes.

### Fractional atomic coordinates and isotropic or equivalent isotropic displacement parameters (Å^2^)

**Table:**

	*x*	*y*	*z*	*U*_iso_*/*U*_eq_	
C1	0.60695 (8)	0.8782 (6)	0.79934 (10)	0.0305 (4)	
H1A	0.586381	0.993978	0.822872	0.046*	
H1B	0.636607	1.007032	0.804205	0.046*	
H1C	0.614950	0.654265	0.818701	0.046*	
C2	0.58026 (7)	0.8460 (5)	0.72475 (10)	0.0225 (4)	
C3	0.53306 (7)	0.9707 (5)	0.69748 (11)	0.0269 (4)	
H3	0.517561	1.081062	0.726518	0.032*	
C4	0.50767 (7)	0.9371 (5)	0.62778 (11)	0.0278 (4)	
H4	0.475869	1.021909	0.611961	0.033*	
C5	0.52935 (6)	0.7800 (5)	0.58267 (10)	0.0239 (4)	
H5	0.512575	0.758554	0.536525	0.029*	
C6	0.57725 (6)	0.6531 (5)	0.60773 (9)	0.0190 (3)	
C7	0.60179 (6)	0.6862 (5)	0.67805 (9)	0.0187 (3)	
C8	0.69229 (6)	0.2303 (5)	0.61767 (9)	0.0186 (3)	
C9	0.65285 (6)	0.4087 (4)	0.62986 (8)	0.0171 (3)	
C10	0.61009 (6)	0.4752 (5)	0.57646 (8)	0.0173 (3)	
C11	0.60642 (6)	0.3678 (5)	0.50886 (9)	0.0213 (4)	
H11	0.578573	0.416290	0.473378	0.026*	
C12	0.64511 (7)	0.1896 (5)	0.49680 (9)	0.0213 (4)	
H12	0.643093	0.114554	0.452561	0.026*	
C13	0.68800 (6)	0.1176 (5)	0.55022 (9)	0.0199 (4)	
C14	0.72687 (7)	−0.0821 (5)	0.53619 (10)	0.0230 (4)	
H14	0.722444	−0.163999	0.491820	0.028*	
C15	0.88208 (8)	0.8160 (5)	0.68439 (9)	0.0265 (4)	
H15A	0.902174	0.905029	0.726989	0.040*	
H15B	0.860603	0.642822	0.693463	0.040*	
H15C	0.862872	0.997972	0.658394	0.040*	
C16	0.91438 (7)	0.6628 (5)	0.64413 (9)	0.0214 (4)	
C17	0.96492 (7)	0.6431 (5)	0.66858 (9)	0.0256 (4)	
H17	0.979856	0.730010	0.711994	0.031*	
C18	0.99471 (7)	0.4977 (5)	0.63077 (10)	0.0270 (4)	
H18	1.028583	0.491116	0.649516	0.032*	
C19	0.97440 (7)	0.3645 (5)	0.56619 (9)	0.0232 (4)	
H19	0.994149	0.267196	0.541288	0.028*	
C20	0.92328 (6)	0.3795 (5)	0.53906 (9)	0.0187 (3)	
C21	0.89409 (6)	0.5292 (5)	0.57814 (8)	0.0179 (3)	
C22	0.80092 (6)	0.2763 (5)	0.42718 (9)	0.0183 (3)	
C23	0.84263 (6)	0.3534 (4)	0.47993 (8)	0.0176 (3)	
C24	0.89000 (6)	0.2655 (5)	0.47573 (8)	0.0180 (3)	
C25	0.89635 (7)	0.0985 (5)	0.41705 (9)	0.0218 (4)	
H25	0.927599	0.043979	0.413640	0.026*	
C26	0.85547 (7)	0.0181 (5)	0.36514 (9)	0.0232 (4)	
H26	0.859196	−0.093655	0.326343	0.028*	
C27	0.80755 (7)	0.1021 (5)	0.36941 (9)	0.0206 (4)	
C28	0.76490 (7)	−0.0010 (5)	0.31623 (9)	0.0254 (4)	
H28	0.769894	−0.116522	0.278565	0.030*	
N1	0.64768 (5)	0.5391 (4)	0.69071 (8)	0.0191 (3)	
N2	0.84527 (5)	0.5107 (4)	0.54173 (7)	0.0187 (3)	
O1	0.73268 (5)	0.1687 (4)	0.67024 (6)	0.0238 (3)	
O2	0.76604 (5)	−0.1520 (4)	0.57967 (7)	0.0276 (3)	
O3	0.75629 (5)	0.3697 (4)	0.43310 (7)	0.0230 (3)	
O4	0.72203 (5)	0.0536 (4)	0.31724 (7)	0.0298 (3)	
H1	0.6700 (9)	0.527 (7)	0.7295 (13)	0.033 (6)*	
H1D	0.7530 (11)	0.035 (9)	0.6508 (16)	0.065 (9)*	
H2	0.8187 (8)	0.600 (6)	0.5531 (12)	0.031 (6)*	
H3A	0.7344 (11)	0.288 (9)	0.3952 (16)	0.063 (9)*	

### Atomic displacement parameters (Å^2^)

**Table:**

	*U*^11^	*U*^22^	*U*^33^	*U*^12^	*U*^13^	*U*^23^
C1	0.0445 (12)	0.0266 (10)	0.0261 (10)	0.0013 (9)	0.0192 (9)	−0.0015 (8)
C2	0.0284 (9)	0.0157 (9)	0.0283 (9)	−0.0017 (7)	0.0160 (8)	0.0015 (7)
C3	0.0298 (10)	0.0167 (9)	0.0411 (11)	0.0003 (7)	0.0213 (9)	0.0011 (8)
C4	0.0212 (9)	0.0196 (9)	0.0443 (12)	0.0017 (7)	0.0120 (8)	0.0058 (8)
C5	0.0203 (8)	0.0193 (9)	0.0310 (10)	−0.0008 (7)	0.0053 (7)	0.0067 (7)
C6	0.0191 (8)	0.0169 (8)	0.0218 (8)	−0.0018 (6)	0.0068 (6)	0.0048 (7)
C7	0.0208 (8)	0.0157 (8)	0.0219 (8)	0.0002 (6)	0.0096 (7)	0.0042 (7)
C8	0.0188 (8)	0.0193 (9)	0.0182 (8)	−0.0015 (7)	0.0059 (6)	0.0015 (6)
C9	0.0181 (7)	0.0172 (8)	0.0170 (8)	−0.0028 (6)	0.0066 (6)	0.0026 (6)
C10	0.0182 (7)	0.0172 (8)	0.0173 (8)	−0.0025 (6)	0.0061 (6)	0.0034 (6)
C11	0.0227 (8)	0.0226 (9)	0.0177 (8)	−0.0034 (7)	0.0041 (6)	0.0027 (7)
C12	0.0268 (9)	0.0218 (9)	0.0158 (8)	−0.0064 (7)	0.0067 (7)	−0.0003 (7)
C13	0.0215 (8)	0.0203 (9)	0.0199 (8)	−0.0039 (7)	0.0089 (6)	−0.0004 (7)
C14	0.0276 (9)	0.0214 (9)	0.0238 (9)	−0.0023 (7)	0.0133 (7)	0.0004 (7)
C15	0.0382 (10)	0.0237 (10)	0.0179 (8)	−0.0029 (8)	0.0083 (7)	−0.0025 (7)
C16	0.0300 (9)	0.0177 (9)	0.0160 (8)	−0.0027 (7)	0.0053 (7)	0.0030 (7)
C17	0.0330 (10)	0.0216 (9)	0.0181 (8)	−0.0055 (8)	−0.0001 (7)	0.0028 (7)
C18	0.0244 (9)	0.0262 (10)	0.0265 (9)	−0.0019 (7)	0.0005 (7)	0.0064 (8)
C19	0.0226 (8)	0.0237 (10)	0.0232 (9)	0.0031 (7)	0.0062 (7)	0.0061 (7)
C20	0.0209 (8)	0.0171 (8)	0.0188 (8)	0.0008 (7)	0.0064 (6)	0.0039 (6)
C21	0.0206 (8)	0.0165 (8)	0.0166 (8)	−0.0004 (6)	0.0052 (6)	0.0034 (6)
C22	0.0214 (8)	0.0180 (8)	0.0166 (8)	0.0002 (7)	0.0068 (6)	0.0031 (6)
C23	0.0222 (8)	0.0174 (8)	0.0143 (8)	0.0004 (7)	0.0070 (6)	0.0016 (6)
C24	0.0206 (8)	0.0171 (8)	0.0171 (8)	0.0028 (6)	0.0064 (6)	0.0040 (6)
C25	0.0245 (9)	0.0225 (9)	0.0203 (8)	0.0045 (7)	0.0092 (7)	0.0024 (7)
C26	0.0325 (10)	0.0220 (9)	0.0165 (8)	0.0030 (8)	0.0093 (7)	−0.0003 (7)
C27	0.0270 (9)	0.0202 (9)	0.0143 (8)	0.0001 (7)	0.0051 (6)	0.0012 (7)
C28	0.0346 (10)	0.0233 (10)	0.0162 (8)	−0.0013 (8)	0.0035 (7)	0.0003 (7)
N1	0.0197 (7)	0.0218 (8)	0.0165 (7)	0.0019 (6)	0.0060 (6)	0.0013 (6)
N2	0.0204 (7)	0.0216 (8)	0.0149 (7)	0.0002 (6)	0.0062 (5)	−0.0005 (6)
O1	0.0187 (6)	0.0312 (8)	0.0203 (6)	0.0047 (5)	0.0034 (5)	−0.0006 (5)
O2	0.0247 (7)	0.0301 (8)	0.0308 (7)	0.0022 (6)	0.0123 (6)	−0.0014 (6)
O3	0.0186 (6)	0.0318 (8)	0.0187 (6)	−0.0013 (5)	0.0051 (5)	−0.0013 (5)
O4	0.0278 (7)	0.0397 (9)	0.0193 (6)	−0.0043 (6)	0.0019 (5)	−0.0011 (6)

### Geometric parameters (Å, º)

**Table:**

C1—C2	1.500 (3)	C15—H15B	0.9600
C1—H1A	0.9600	C15—H15C	0.9600
C1—H1B	0.9600	C16—C17	1.382 (3)
C1—H1C	0.9600	C16—C21	1.404 (2)
C2—C3	1.387 (3)	C17—C18	1.404 (3)
C2—C7	1.404 (2)	C17—H17	0.9300
C3—C4	1.405 (3)	C18—C19	1.380 (3)
C3—H3	0.9300	C18—H18	0.9300
C4—C5	1.376 (3)	C19—C20	1.401 (2)
C4—H4	0.9300	C19—H19	0.9300
C5—C6	1.402 (2)	C20—C21	1.417 (2)
C5—H5	0.9300	C20—C24	1.440 (2)
C6—C7	1.411 (2)	C21—N2	1.379 (2)
C6—C10	1.438 (2)	C22—O3	1.350 (2)
C7—N1	1.378 (2)	C22—C23	1.392 (2)
C8—O1	1.354 (2)	C22—C27	1.410 (2)
C8—C9	1.393 (2)	C23—N2	1.379 (2)
C8—C13	1.409 (2)	C23—C24	1.408 (2)
C9—N1	1.378 (2)	C24—C25	1.410 (2)
C9—C10	1.410 (2)	C25—C26	1.371 (3)
C10—C11	1.409 (2)	C25—H25	0.9300
C11—C12	1.374 (3)	C26—C27	1.420 (3)
C11—H11	0.9300	C26—H26	0.9300
C12—C13	1.417 (2)	C27—C28	1.439 (2)
C12—H12	0.9300	C28—O4	1.237 (2)
C13—C14	1.438 (3)	C28—H28	0.9300
C14—O2	1.244 (2)	N1—H1	0.87 (2)
C14—H14	0.9300	N2—H2	0.91 (2)
C15—C16	1.505 (3)	O1—H1D	0.94 (3)
C15—H15A	0.9600	O3—H3A	0.90 (3)
			
C2—C1—H1A	109.5	H15A—C15—H15C	109.5
C2—C1—H1B	109.5	H15B—C15—H15C	109.5
H1A—C1—H1B	109.5	C17—C16—C21	115.81 (17)
C2—C1—H1C	109.5	C17—C16—C15	123.33 (17)
H1A—C1—H1C	109.5	C21—C16—C15	120.86 (16)
H1B—C1—H1C	109.5	C16—C17—C18	122.89 (17)
C3—C2—C7	115.79 (17)	C16—C17—H17	118.6
C3—C2—C1	122.54 (17)	C18—C17—H17	118.6
C7—C2—C1	121.68 (17)	C19—C18—C17	120.89 (18)
C2—C3—C4	122.71 (18)	C19—C18—H18	119.6
C2—C3—H3	118.6	C17—C18—H18	119.6
C4—C3—H3	118.6	C18—C19—C20	118.34 (18)
C5—C4—C3	120.74 (17)	C18—C19—H19	120.8
C5—C4—H4	119.6	C20—C19—H19	120.8
C3—C4—H4	119.6	C19—C20—C21	119.64 (16)
C4—C5—C6	118.62 (18)	C19—C20—C24	133.72 (17)
C4—C5—H5	120.7	C21—C20—C24	106.64 (15)
C6—C5—H5	120.7	N2—C21—C16	128.23 (16)
C5—C6—C7	119.64 (17)	N2—C21—C20	109.32 (15)
C5—C6—C10	133.48 (17)	C16—C21—C20	122.43 (16)
C7—C6—C10	106.86 (15)	O3—C22—C23	119.39 (15)
N1—C7—C2	128.29 (17)	O3—C22—C27	122.90 (16)
N1—C7—C6	109.21 (15)	C23—C22—C27	117.71 (16)
C2—C7—C6	122.50 (16)	N2—C23—C22	128.21 (16)
O1—C8—C9	119.60 (15)	N2—C23—C24	110.28 (15)
O1—C8—C13	122.58 (16)	C22—C23—C24	121.50 (16)
C9—C8—C13	117.81 (16)	C23—C24—C25	120.38 (16)
N1—C9—C8	128.95 (16)	C23—C24—C20	105.83 (15)
N1—C9—C10	109.92 (15)	C25—C24—C20	133.78 (16)
C8—C9—C10	121.13 (16)	C26—C25—C24	118.57 (16)
C11—C10—C9	120.79 (16)	C26—C25—H25	120.7
C11—C10—C6	133.37 (16)	C24—C25—H25	120.7
C9—C10—C6	105.84 (15)	C25—C26—C27	121.34 (17)
C12—C11—C10	118.20 (16)	C25—C26—H26	119.3
C12—C11—H11	120.9	C27—C26—H26	119.3
C10—C11—H11	120.9	C22—C27—C26	120.48 (16)
C11—C12—C13	121.50 (16)	C22—C27—C28	118.85 (17)
C11—C12—H12	119.3	C26—C27—C28	120.62 (17)
C13—C12—H12	119.3	O4—C28—C27	124.41 (18)
C8—C13—C12	120.56 (16)	O4—C28—H28	117.8
C8—C13—C14	119.52 (16)	C27—C28—H28	117.8
C12—C13—C14	119.89 (16)	C9—N1—C7	108.16 (14)
O2—C14—C13	124.09 (17)	C9—N1—H1	124.4 (16)
O2—C14—H14	118.0	C7—N1—H1	127.5 (16)
C13—C14—H14	118.0	C23—N2—C21	107.92 (15)
C16—C15—H15A	109.5	C23—N2—H2	123.7 (15)
C16—C15—H15B	109.5	C21—N2—H2	128.2 (15)
H15A—C15—H15B	109.5	C8—O1—H1D	104.5 (18)
C16—C15—H15C	109.5	C22—O3—H3A	105.7 (19)
			
C7—C2—C3—C4	0.8 (3)	C18—C19—C20—C21	0.0 (3)
C1—C2—C3—C4	−179.15 (19)	C18—C19—C20—C24	−178.91 (19)
C2—C3—C4—C5	−0.9 (3)	C17—C16—C21—N2	179.02 (18)
C3—C4—C5—C6	0.2 (3)	C15—C16—C21—N2	−0.9 (3)
C4—C5—C6—C7	0.5 (3)	C17—C16—C21—C20	0.7 (3)
C4—C5—C6—C10	178.98 (19)	C15—C16—C21—C20	−179.26 (17)
C3—C2—C7—N1	−179.66 (18)	C19—C20—C21—N2	−179.13 (16)
C1—C2—C7—N1	0.3 (3)	C24—C20—C21—N2	0.0 (2)
C3—C2—C7—C6	0.0 (3)	C19—C20—C21—C16	−0.5 (3)
C1—C2—C7—C6	179.92 (17)	C24—C20—C21—C16	178.64 (16)
C5—C6—C7—N1	179.06 (16)	O3—C22—C23—N2	2.3 (3)
C10—C6—C7—N1	0.2 (2)	C27—C22—C23—N2	−177.78 (17)
C5—C6—C7—C2	−0.6 (3)	O3—C22—C23—C24	−179.11 (16)
C10—C6—C7—C2	−179.45 (16)	C27—C22—C23—C24	0.8 (3)
O1—C8—C9—N1	−0.6 (3)	N2—C23—C24—C25	179.47 (16)
C13—C8—C9—N1	178.76 (17)	C22—C23—C24—C25	0.6 (3)
O1—C8—C9—C10	−179.52 (16)	N2—C23—C24—C20	−0.3 (2)
C13—C8—C9—C10	−0.2 (3)	C22—C23—C24—C20	−179.20 (16)
N1—C9—C10—C11	179.77 (16)	C19—C20—C24—C23	179.2 (2)
C8—C9—C10—C11	−1.1 (3)	C21—C20—C24—C23	0.18 (19)
N1—C9—C10—C6	−0.63 (19)	C19—C20—C24—C25	−0.6 (4)
C8—C9—C10—C6	178.49 (16)	C21—C20—C24—C25	−179.59 (19)
C5—C6—C10—C11	1.2 (4)	C23—C24—C25—C26	−1.3 (3)
C7—C6—C10—C11	179.77 (19)	C20—C24—C25—C26	178.46 (19)
C5—C6—C10—C9	−178.36 (19)	C24—C25—C26—C27	0.5 (3)
C7—C6—C10—C9	0.24 (19)	O3—C22—C27—C26	178.33 (17)
C9—C10—C11—C12	1.6 (3)	C23—C22—C27—C26	−1.6 (3)
C6—C10—C11—C12	−177.90 (19)	O3—C22—C27—C28	−4.1 (3)
C10—C11—C12—C13	−0.8 (3)	C23—C22—C27—C28	175.89 (16)
O1—C8—C13—C12	−179.70 (16)	C25—C26—C27—C22	1.0 (3)
C9—C8—C13—C12	1.0 (3)	C25—C26—C27—C28	−176.49 (18)
O1—C8—C13—C14	2.4 (3)	C22—C27—C28—O4	0.3 (3)
C9—C8—C13—C14	−176.92 (16)	C26—C27—C28—O4	177.80 (19)
C11—C12—C13—C8	−0.5 (3)	C8—C9—N1—C7	−178.24 (18)
C11—C12—C13—C14	177.38 (17)	C10—C9—N1—C7	0.8 (2)
C8—C13—C14—O2	−3.4 (3)	C2—C7—N1—C9	179.03 (18)
C12—C13—C14—O2	178.69 (18)	C6—C7—N1—C9	−0.6 (2)
C21—C16—C17—C18	−0.4 (3)	C22—C23—N2—C21	179.13 (17)
C15—C16—C17—C18	179.59 (18)	C24—C23—N2—C21	0.4 (2)
C16—C17—C18—C19	−0.1 (3)	C16—C21—N2—C23	−178.75 (18)
C17—C18—C19—C20	0.3 (3)	C20—C21—N2—C23	−0.3 (2)

### Hydrogen-bond geometry (Å, º)

**Table:**

*D*—H···*A*	*D*—H	H···*A*	*D*···*A*	*D*—H···*A*
C14—H14···O3^i^	0.93	2.50	3.254 (2)	138
N1—H1···O4^ii^	0.87 (2)	2.00 (2)	2.862 (2)	174 (2)
O1—H1*D*···O2	0.94 (3)	1.74 (3)	2.602 (2)	151 (3)
N2—H2···O2^iii^	0.91 (2)	1.97 (2)	2.879 (2)	173 (2)
O3—H3*A*···O4	0.90 (3)	1.78 (3)	2.595 (2)	150 (3)

Symmetry codes: (i) *x*, *y*−1, *z*; (ii) *x*, −*y*+1/2, *z*+1/2; (iii) *x*, *y*+1, *z*.
